# MicroRNA-212-5p and its target PAFAH1B2 suppress vascular proliferation and contraction *via* the downregulation of RhoA

**DOI:** 10.1371/journal.pone.0249146

**Published:** 2021-03-24

**Authors:** Gwi Ran Kim, Tingwei Zhao, Hae Jin Kee, Seung-Jung Kee, Myung Ho Jeong

**Affiliations:** 1 Heart Research Center of Chonnam National University Hospital, Gwangju, Republic of Korea; 2 Hypertension Heart Failure Research Center, Chonnam National University Hospital, Gwangju, Republic of Korea; 3 Department of Laboratory Medicine, Chonnam National University, Medical School and Hospital, Gwangju, Republic of Korea; 4 Department of Cardiology, Chonnam National University Medical School, Gwangju, Republic of Korea; Mayo Clinic Minnesota, UNITED STATES

## Abstract

Vascular remodeling and contraction contribute to the development of hypertension. We investigated the role of miR-212-5p and its downstream target in vascular smooth muscle cell (VSMC) proliferation, migration, and contraction. MicroRNA microarray and PCR analyses showed that miR-212-5p expression was increased with angiotensin II treatment in vivo and in vitro. Moreover, miR-212-5p mimic treatment attenuated and miR-212-5p inhibitor treatment increased VSMC proliferation and migration. Additionally, miR-212-5p mimic treatment suppressed VSMC contraction and related gene expression [Ras homolog gene family member A (RhoA) and Rho-associated protein kinase 2], while miR-212-5p inhibitor treatment exerted opposite effects. Bioinformatics analysis revealed that platelet-activating factor acetylhydrolase 1B2 (PAFAH1B2) is a target of miR-212-5p. miR-212-5p mimic treatment significantly reduced and miR-212-5p inhibitor treatment increased PAFAH1B2 expression. Furthermore, PAFAH1B2 expression was decreased in angiotensin II-treated aortic tissues and VSMCs. PAFAH1B2 was ubiquitously expressed in most adult rat tissues. In the vasculature, PAFAH1B2 was only distributed in the cytoplasm. PAFAH1B2 overexpression decreased A10 cell proliferation, while PAFAH1B2 knockdown increased A10 cell proliferation and *cyclin D1* mRNA levels. PAFAH1B2 knockdown stimulated VSMC contraction and RhoA expression. These results suggest that miR-212-5p and PAFAH1B2 are novel negative regulators of VSMC proliferation, migration, and contraction in hypertension.

## Introduction

MicroRNA (miRNA) is a short, 18–25-nucleotide-long, single-stranded non-coding RNA identified in many eukaryotes [1]. miRNA regulates gene expression through post-transcriptional regulation [2] and plays a major role in inhibiting target mRNA expression [3]. miRNAs are abnormally overexpressed or downregulated in many different pathological diseases, including metabolic disease, diabetes, cardiac hypertrophy, heart failure, and cancer etc. [4,5]. Recent research has shown that a variety of miRNAs are associated with essential hypertensive animal models or human population [6–8]. Especially, vascular smooth muscle cell (VSMC) proliferation and migration-related miRNAs have been reported [9]. For example, miR-93/target Mitofusin-2 (MFN2) [10], miR-149-5p/target Histone deacetylase 4 (HDAC4) [11], miR-612/target RAC-beta serine/threonine-protein kinase (AKT2) [12], miR-145/target ROCK1 [13], miR-362-3p/target A disintegrin and metalloproteinase with thrombospondin motifs 1 (ADAMTS1) [14], miR-22-3p or miR-24/target High mobility group box 1 (HMGB1) [15], and miR-379/target Insulin-like growth factor 1 (IGF-1) [16] were shown to be involved in VSMC proliferation and migration. However, little is known about the functional relevance of miRNA in vascular contraction.

Angiotensin II is a strong vasoconstrictor peptide that binds to angiotensin receptor type I (AT1) and induces vascular contraction and arterial remodeling as well as VSMC growth. Multiple studies, including ours, have demonstrated that in animal models of NG nitroarginine methyl ester (L-NAME)- or angiotensin II-induced hypertension, as well as in spontaneously hypertensive rats, blood pressure is markedly increased [17,18].

We hypothesized that unknown miRNAs may regulate arterial remodeling and vasoconstriction in hypertension. Here, we investigated novel miRNAs involved in the regulation of hypertension using miRNA microarrays. Results showed that members of the miR-34c family (miR34c-5p, miR34c-3p, and miR34b-3p) were most highly expressed, followed by miR-132-5p/3p, miR-381-3p, and miR-409-5p in the aorta of angiotensin II-treated mice. Studies on miR-34c, miR-132-5p, and miR-132-3p have been previously published [19–22]. It has also been reported that miR-212 expression is increased or decreased in various cancers, suggesting its potential role as a biomarker [23]. For example, miR-212-5p overexpression inhibits cell migration and invasion of triple-negative breast cancer cells *in vitro* by downregulating paired related homeobox 2 (Prrx2) expression [24]. Furthermore, miR-212-5p prevents dopaminergic neuron death in the pathogenesis of Parkinson’s disease by inhibiting sirtuin 2 (SIRT2) expression and activity [25]. Therefore, we hypothesized that miR-212-5p also plays a beneficial role in vascular diseases.

In this study, we investigated the role of miR-212-5p and its downstream target, platelet-activating factor acetylhydrolase 1B2 (PAFAH1B2), in the regulation of VSMC proliferation, migration, and contraction.

## Methods

### Reagents and antibodies

Angiotensin II was purchased from Calbiochem (Merck Millipore, MA, USA). miRNA 212-5p mimic, mimic control, and inhibitor control were purchased from Bioneer (Daejeon, South Korea). miRNA 212-5p primer was purchased from Applied Biosystems (Waltham, MA, USA). The miRNA PCR kit and miRNA 212-5p inhibitor were purchased from Thermo Fisher Scientific (Waltham, MA, USA). Anti-Glyceraldehyde-3-Phosphate Dehydrogenase (GAPDH) (sc-32233), anti-PAFAH1B2 (sc-393217), anti-ACY1 (sc-374258), anti-RhoA (sc-418), anti-ROCK2 (sc-398519), anti-β-actin (sc-47778), and anti-Lamin B (sc-6216) antibodies were purchased from Santa Cruz Biotechnology (Dallas, TX, USA). MFSD2A (105399) antibody was purchased from Abcam (Cambridge, UK). Mouse *pCMV6-Pafah1b2* clone was obtained from Korea Human Gene Bank, Medical Genomics Research Center (KRIBB; Daejeon, Korea).

### Hypertension animal model and blood pressure measurement

All animal procedures were approved by the Animal Experimental Committee of Chonnam National University Medical School (CNUHIACUC-20003) and were carried out in accordance with the Guide for the Care and Use of Laboratory Animals (National Institutes of Health, USA; 8^th^ edition, 2011).

CD-1 male mice were purchased from Orient Bio Inc. (Gyeonggi-do, South Korea). Mice were anesthetized via intraperitoneal injection of ketamine (120 mg∙kg^–1^) and xylazine (6.2 mg∙kg^–1^). Angiotensin II (n = 12) was subcutaneously infused to mice via an ALZET® osmotic pump (DURECT Corporation. Cupertino, CA, USA). Systolic blood pressure was then measured in conscious mice by the tail-cuff method (BP-2000; Zur Schoenen Aussicht, Zoellnitz, Germany). For blood pressure measurements, animals were trained three times a week. Two weeks after angiotensin II infusion, mice were sacrificed via carbon dioxide inhalation.

### Arterial wall thickness

Aorta tissues were fixed in 4% paraformaldehyde overnight, embedded in paraffin, and cut into 3 μm thick sections. After hematoxylin and eosin (H&E) staining, the aortic wall thickness was measured using the NIS Elements Software (n = 6; Nikon, Tokyo, Japan).

### RNA quality check

Total RNA was isolated from mouse aorta tissues. For quality control, RNA purity and integrity were evaluated by calculating the optical density (OD) 260/280 ratio and analyzed using the Agilent 2100 Bioanalyzer (Agilent Technologies, Santa Clara, CA, USA).

### Affymetrix miRNA microarray

The Affymetrix Genechip miRNA 4.0 array process was carried out according to the manufacturer’s protocol. Labeling of RNA samples (1 μg) was performed with the FlashTag^TM^ Biotin RNA Labeling Kit (Genisphere, Hatfield, PA, USA). The labeled RNA was quantified, fractionated, and hybridized to the miRNA microarray according to the standard procedures provided by the manufacturer. The labeled RNA was heated to 99°C for 5 min and then to 45°C for 5 min. RNA-array hybridization was performed with agitation at 60 rotations/min for 16 h at 48°C on the Affymetrix® 450 Fluidics Station (Santa Clara, CA, USA). The chips were washed and stained in the GeneChip Fluidics Station 450 (Affymetrix, Santa Clara, CA, USA). The chips were then scanned with an Affymetrix GCS 3000 scanner (Affymetrix, Santa Clara, CA, USA), and signal values were computed using the Affymetrix® GeneChip Command Console software.

### Analysis of microarray data and miRNA target genes

miRNAs with increased expression in the angiotensin II group compared to the sham group were identified. miR-212-5p and miR-212-3p were selected, and their expression was validated by PCR. MiRBase Target database (www.mirbase.org) or TargetScan (www.targetscan.org) was used to predict the target genes of miR-212-5p. Candidate target genes were then evaluated by real-time reverse transcription-PCR (RT-PCR).

### Cell culture

VSMCs were isolated from the aortic media of male rats by the collagenase dissociation method, as described previously [26]. VSMCs were maintained in Dulbecco’s modified Eagle’s medium (DMEM) containing 10% fetal bovine serum (FBS) with high glucose in an incubator at 5% CO_2_. VSMCs between passage 4 and 7 were used for all experiments.

A10 cells derived from the thoracic aorta of embryonic rat (American Type Culture Collection, Manassas, VA, USA) were cultured in DMEM with 10% FBS and subsequently used for overexpression and proliferation experiments.

### Cell transfection

For overexpression or inhibition of miRNA, VSMCs were transfected with control mimic/inhibitor (100 nM), miR-212-5p mimic (100 nM), or miR-212-5p inhibitor (100 nM) for 2 days using RNAiMAX (Invitrogen, Waltham, MA, USA) according to the manufacturer’s instructions. For knockdown of PAFAH1B2, VSMCs were transfected with control or PAFAH1B2 small interfering RNA (siRNA) using RNAiMAX. Target gene knockdown was confirmed by RT-PCR and western blot analysis. For overexpression of PAFAH1B2, A10 cells or 293T cells (Korean Cell Line Bank, Seoul, South Korea) were transfected with empty vector or *pCMV6-pafah1b2* for 2 days using Lipofectamine with PLUS reagents according to the manufacturer’s instructions (Thermo Fisher Scientific, Waltham, MA, USA).

### Cell proliferation

VSMCs or A10 cells were transfected with control, miR-212-5p mimic, miR-212-5p inhibitor, PAFAH1B2 siRNA, or *pCMV6-pafah1b2* for 2 days using RNAiMAX (Invitrogen) or Lipofectamine according to the manufacturer’s instructions. The cells were then incubated with 3-(4,5-dimethylthiazol-2-yl)-2,5-diphenyltetrazolium bromide (MTT) solution for 2 h. The insoluble formazan crystals were dissolved after dimethyl sulfoxide (DMSO) addition, and the absorbance was measured at 570 nm. For cell counting, cells were trypsinized and counted by the trypan blue exclusion assay.

### Bromodeoxyuridine (BrdU) incorporation

After transfection, VSMCs were incubated with BrdU (10 μM) for 75 min. Cells were then fixed with 70% ethanol for 45 min and denatured with 1 N HCl for 20 min. Next, cells were blocked with normal goat serum and incubated with BrdU antibody for 1 h, followed by incubation with rabbit anti-mouse secondary antibody (1:400, 1 h). The slides were mounted with an antifade reagent containing 4′,6-diamidine-2′-phenylindole dihydrochloride (DAPI). BrdU incorporation was expressed as the percentage of BrdU-positive cells in the total number of cells.

### In vitro scratch assay

VSMCs were seeded on 12-well plates and grown until they reached confluency. The plates were scratched with a sterile pipette tip to remove cells. After 17 or 24 h, the cells that migrated into the scratch area were captured under a microscope (Nikon Eclipse Ti-U; Nikon, Japan). The migration rate was calculated as follows: Migration (%) = (Area_0_–Area_t_)/Area_0_ × 100%, where Area_0_ is the area at 0 h and Area_t_ is the area at indicated hour. Data are shown as the percentage of wound area closure (average of 3 independent microscopic fields for each independent experiment).

### Cell contraction assay

VSMCs transfected with control, miR-212-5p mimic, miR-212-5p inhibitor, or PAFAH1B2 siRNA were mixed with collagen gel working solution according to the manufacturer’s instructions (Cell Biolabs Inc., San Dieo, CA, USA). The cell-collagen mixture was added into a 24-well plate and incubated at 37°C for 1 h for collagen polymerization. Culture medium without 10% FBS was added atop collagen gel lattice. The next day, cells were treated with U46619 (300 nM), and then, collagen gels were released using a sterile spatula. The change in collagen gel size was measured using a ruler up to 2 days. The percentage of contraction was calculated by the following equation: Contraction (%) = (Area_0d_–Area_2d_)/Area_0_ × 100%, where Area_0d_ is the area at day 0, and Area_2d_ is the area of at the indicated day.

### RT-PCR analysis

Total RNA was isolated from the aorta tissues using TRIzol reagent (Invitrogen Life Technologies, Carlsbad, CA, USA), and 1 μg of RNA was used for the reverse transcription reaction with TOPscript RT DryMIX (Enzynomics, Daejeon, South Korea). mRNA levels were quantified using a SYBR Green PCR kit (Enzynomics). The PCR primers used in this study are listed in Table 1.

**Table 1:** Primers for RT-PCR.

Gene	Primer sequence (5′ to 3′)
*GAPDH (rat)*	F: AACCCATCACCATCTTCCAGGAGC
	R: ATGGACTGTGGTCATGAGCCCTTC
*Plxna2 (rat)*	F: ACCTGGGCTTGGACTTCTCT
	R: ATACTGCTGGTGGGACTTGG
*Tgfbr3 (rat)*	F: GCCTTGATGGAGAGCTTCAC
	R: GCCAGTCTCTCCGTCTTCAG
*Rasl10b(rat)*	F: CTGCTGCTTTGACAGCTTTG
	R: CTGAAGAGCAGCAGGATGTG
*Faah (rat)*	F: ACTTTGTGGATCCCTGCTTG
	R: TCTCATGCTGCAGTTTCCAC
*Tecanc2 (rat)*	F: AACCATCTGGACAGACCCTCT
	R: CGGTAGGGCCTGTTAATGAG
*Ralgds (rat)*	F: TCAACGGGGTCATCTACTCC
	R: CCAGCTTCTCCAGAGTACCG
*Ccdc117 (rat)*	F: GCCTTTCTGTGCCTAGCATC
	R: TGGGAGGTGGCTAACATTTC
*Mfsd2a (rat)*	F: CCAGGTGAAGAAGGAACCAA
	R: CCCACGAAAAGGATAATGGA
*Acy1(rat)*	F: GGGTGGTGTGGCCTATAATG
	R: CAGGGGTCCGTATCATCAGT
*Pafah1b2 (rat)*	F: GGTGAACCAGCTTCTCAAGG
	R: TGGTCTGCTTCTCCTCTGGT
*Cyclin D1 (rat)*	F: CTGCATGTTCGTGGCCTCTA
	R: CCTCTGGCATTTTGGAGAGG
*RhoA (rat)*	F: AGGACCAGTTCCCAGAGGTT
	R: ACTATCAGGGCTGTCGATGG
*ROCK2 (rat)*	F: TGCTGGATGGCTTAAATTCC
	R: TCACCAAAAGCACCTCTTCC
*GAPDH (mouse)*	F: GCATGGCCTTCCGTGTTCCT
	R: CCCTGTTGCTGTAGCCGTATTCAT
*Pafah1b2 (mouse)*	F: AGAATGCCAAGGTGAACCAG
	R: ATAGCCCCCTCCTGTGAGAT

### Western blotting

Total protein was extracted from the cells using radioimmunoprecipitation assay (RIPA) lysis buffer (150 mM NaCl, 1% Triton X-100, 1% sodium deoxycholate, 50 mM Tris-HCl pH 7.5, 2 mM EDTA, 1 mM PMSF, 1 mM DTT, 1 mM Na_3_VO_4_, and 5 mM NaF) containing a protease inhibitor cocktail (Calbiochem, EMD Millipore, Billerica, MA, USA). Proteins were separated by sodium dodecyl sulfate-polyacrylamide gel electrophoresis (SDS-PAGE) and then transferred onto polyvinylidene difluoride membranes. The membranes were blocked with 5% skim milk in Tris-buffered saline with Tween® 20 buffer (20 mM Tris, 200 mM NaCl, and 0.04% Tween® 20) for 1 h at 25°C, and then incubated overnight at 4°C with the indicated primary antibodies. Following this, membranes were incubated with anti-rabbit or anti-mouse horseradish-peroxidase-conjugated secondary antibodies (1:5000) for 1 h at 25°C. Protein bands were visualized using Immobilon Western detection reagents (EMD Millipore, Burlington, MA, USA). The Bio-ID software (Vilber Lourmat, Eberhardzell, Germany) was used for protein quantification.

To analyze the distribution of PAFAH1B2 in the tissue blots, rat brain, heart, aorta, kidney, lung, liver, stomach, skeletal muscle, spleen, testis, and seminal vesicle samples were prepared from 10-week-old male rats using RIPA lysis buffer.

### Isolation of nuclear and cytoplasmic extracts

Cytoplasmic and nuclear extracts were isolated using buffer A (10 mM HEPES, pH 7.9, 10 mM KCl, 10 mM EDTA, 1 mM DTT, 0.4% IGEPAL CA630, and protease inhibitor) and buffer B (20 mM HEPES, pH 7.9, 400 mM NaCl, 1 mM EDTA, 10% glycerol, 1 mM DTT, and protease inhibitor). Lamin B and GAPDH were used as loading controls for nuclear and cytoplasmic extracts, respectively.

### Statistical analysis

All data are expressed as mean ± standard error of the mean. Comparison between 2 groups was performed by Student’s *t*-test. A value of *P* < 0.05 was considered statistically significant.

## Results

### miR-212-5p expression increases in angiotensin II-treated hypertensive mice and VSMCs

**Fig 1:**
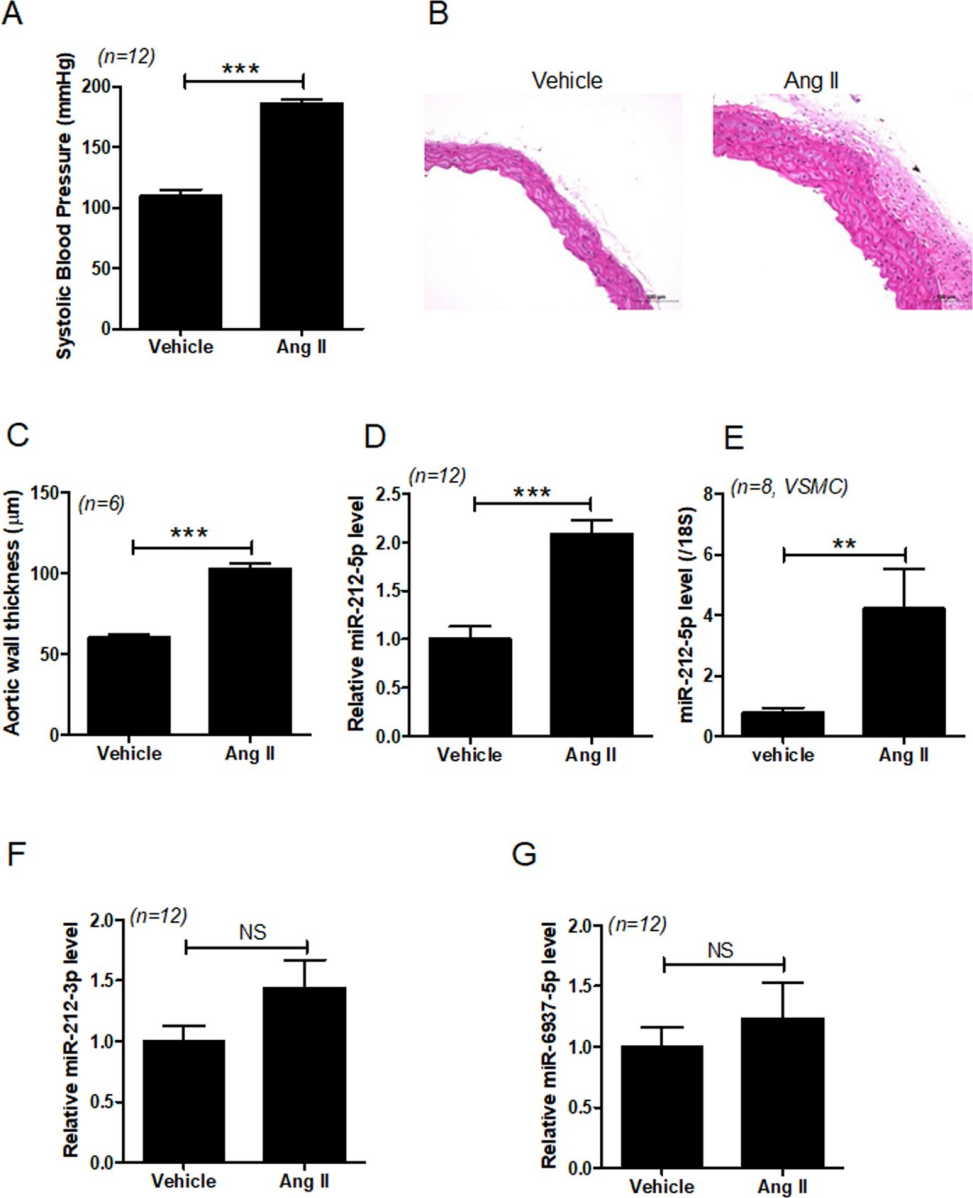
miR 212-5p expression is increased in aortic tissues from angiotensin II-treated mice and VSMCs. **(A**) Systolic blood pressure was measured in vehicle- and angiotensin II-treated mice (n = 12 per group). **(B)** Representative images of H&E staining of aortic tissues (n = 6 per group). Scale bar = 100 μm. (**C**) Quantified aortic wall thickness (n = 6 per group) in vehicle- and angiotensin II-treated mice. (**D**) Aortic miR-212-5plevels were evaluated using RT-PCR. ***P < 0.001 versus the vehicle-treated group. (**E**) VSMCs were serum-starved and then treated with angiotensin II (1 μM) for 1 day. miR-212-5p levels were evaluated by RT-PCR. **P < 0.01 versus the vehicle-treated group. (**F,G**) Aortic miR-212-3p and miR-6937-5p levels were evaluated using RT-PCR. NS indicates not significant.

We observed that treatment with angiotensin II increased the blood pressure and aortic wall thickness in mice (Fig 1A–1C). To identify new miRNAs that regulate hypertension, we performed Affymetrix Genechip miRNA 4.0 array. Several miRNAs whose expression was increased or reduced in angiotensin II-treated mice were selected. Among these miRNAs, we found that miR-212-5p expression was significantly increased in the aorta of angiotensin II-treated mice and angiotensin II-treated VSMCs (Fig 1D and 1E). However, miR-212-3p level was not significantly increased in the aortic tissues from angiotensin II-treated mice (Fig 1F). Additionally, microRNA microarray showed that miR-6937-5p level was downregulated in angiotensin II-treated mice. However, RT-PCR revealed that miR-6937-5p level was not significantly reduced in angiotensin II-treated mice compared to that in vehicle-treated mice (Fig 1G). Hence, we further investigated the role of miR-212-5p in hypertension.

### miR-212-5p regulates VSMC proliferation and migration

**Fig 2:**
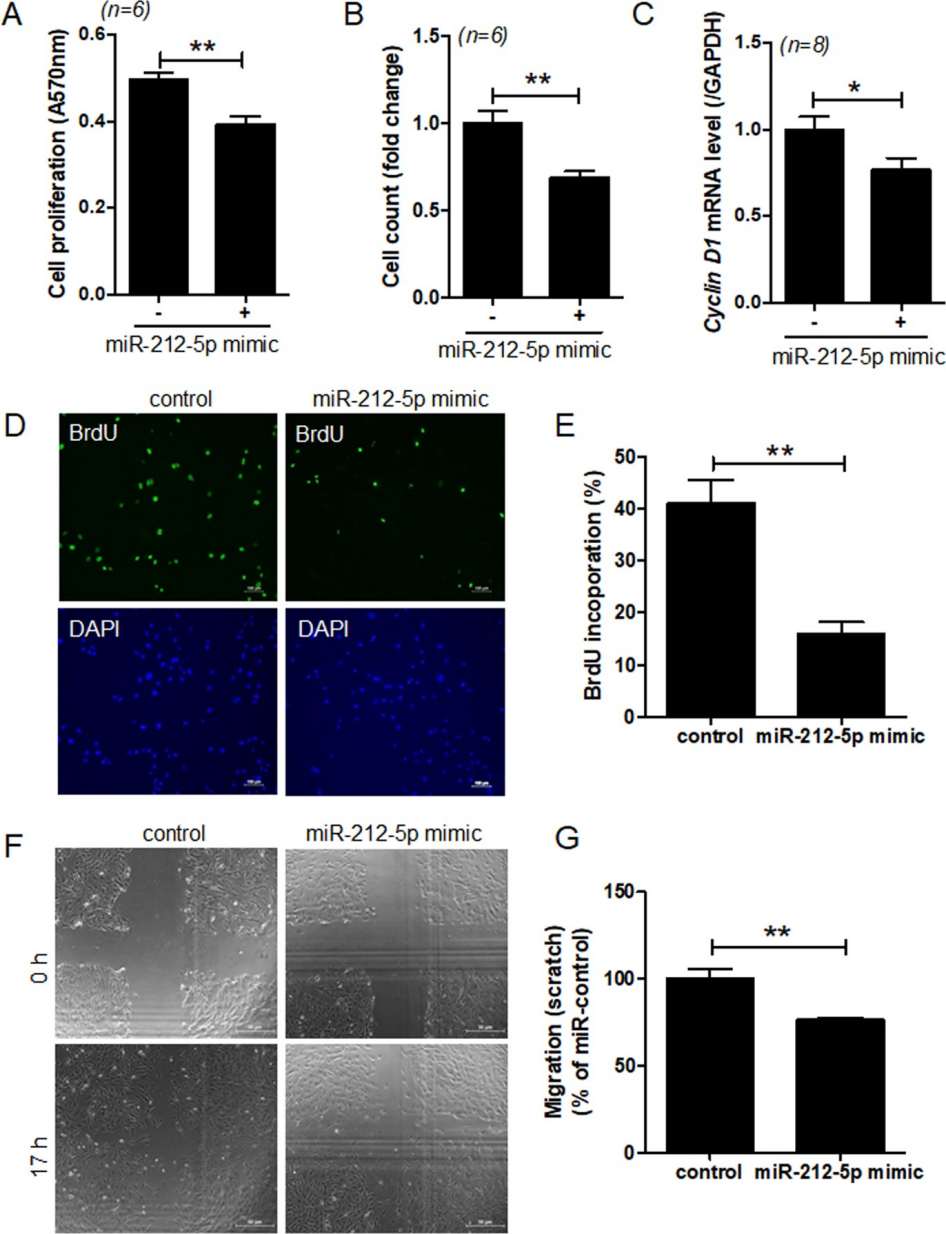
miR-212-5p overexpression attenuates VSMC proliferation and migration. **(A)** VSMCs were transfected with control or miR-212-5p mimic for 2 days. Cell proliferation was determined by the MTT assay. **P < 0.01 versus the control mimic group. (**B)** Direct cell counting showing in vitro proliferation of VSMCs transfected with miR-212-5p mimic. **P < 0.01 versus the control mimic group. (**C**) After transfection of VSMCs with miR-212-5p mimic, cyclin D1 transcript levels were determined by RT-PCR. *P < 0.05 versus the control mimic group. (**D,E**) VSMCs transfected with miR-212-5p mimic were incubated with BrdU and then immunostained with an anti-BrdU antibody. Scale bar = 100 μm. The number of BrdU-positive cells was quantified. **P < 0.01 versus the control mimic group. (**F,G**) VSMCs were transfected with control or miR-212-5p mimic, and cell migration was assessed for 15~24 h by the scratch-wound assay. **P < 0.01 versus the control mimic group.

We hypothesized that angiotensin II-induced miR-212-5p expression is involved in cell proliferation and migration. Two days after miR-212-5p mimic transfection, VSMC proliferation was significantly reduced compared to that after control mimic transfection (Fig 2A). Direct cell counting results were the same for both groups (Fig 2B). Moreover, miR-212-5p mimic treatment significantly decreased *cyclin D1* mRNA levels compared to that in control mimic treatment (Fig 2C). Cell proliferation was also determined using the BrdU incorporation assay. As shown in Fig 2D, BrdU staining was observed in the nucleus of VSMCs. However, miR-212-5p mimic transfection significantly reduced BrdU incorporation relative to control mimic transfection (Fig 2E). We next examined whether miR-212-5p overexpression affects VSMC migration, using the scratch assay. Results showed that transfection of VSMCs with miR-212-5p mimic attenuated cell migration (Fig 2F and 2G).

**Fig 3:**
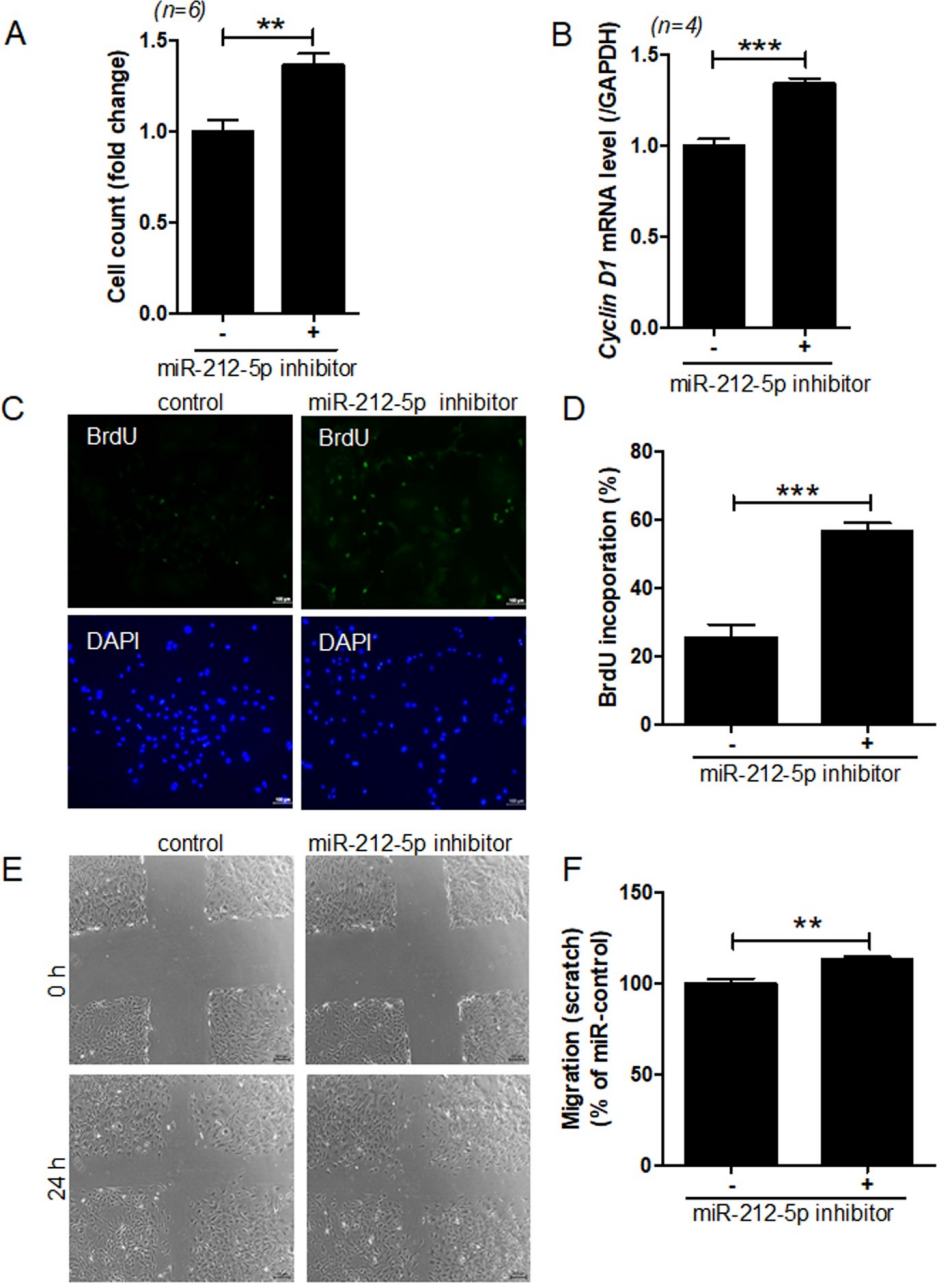
miR-212-5p inhibition increases VSMC proliferation and migration. VSMCs were transfected with control or miR-212-5p inhibitor for 2 days. (**A**) Cell proliferation was determined by the MTT assay. **P < 0.01 versus the control inhibitor group. (B) After transfection of VSMCs with miR-212-5p inhibitor, cyclin D1 transcript levels were determined by RT-PCR. ***P < 0.001 versus the control inhibitor group. (**C,D**) VSMCs transfected with miR-212-5p inhibitor were incubated in the presence of BrdU and then immunostained with an anti-BrdU antibody. Scale bar = 100 μm. The number of BrdU-positive cells was quantified. ***P < 0.001 versus the control inhibitor group. (**E,F**) VSMCs were transfected with control or miR-212-5p inhibitor, and cell migration was assessed for 15~24 h by the scratch-wound assay. **P < 0.01 versus the control inhibitor group.

Contrary to miR-212-5p mimic, treatment with miR-212-5p inhibitor increased cell proliferation (Fig 3A), *cyclin D1* mRNA level (Fig 3B), BrdU incorporation (Fig 3C and 3D), and migration (Fig 3E and 3F).

### miR-212-5p regulates cell contraction through the RhoA-ROCK axis

**Fig 4:**
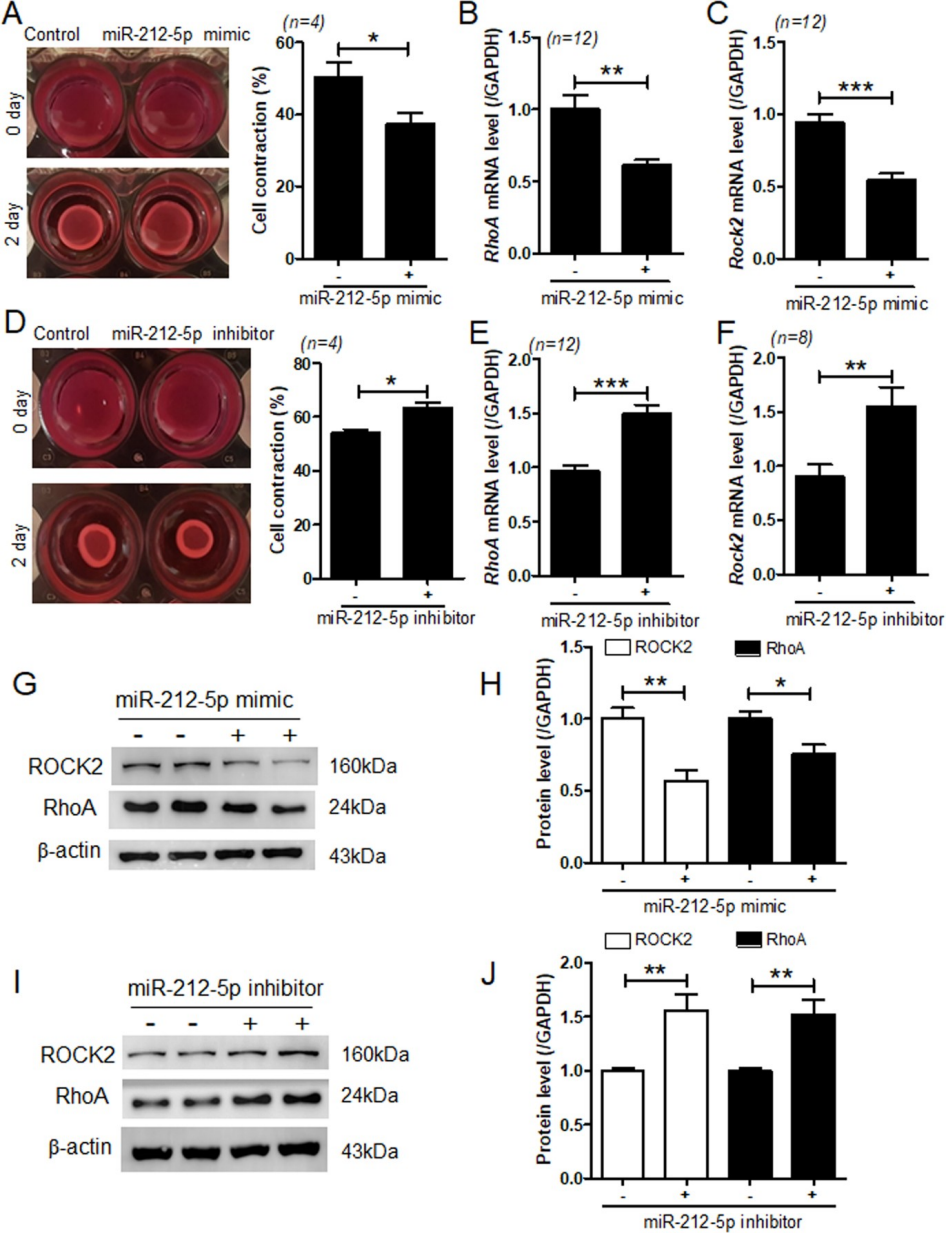
miR-212-5p regulates VSMC contraction. **(A)** VSMCs were transfected with control or miR-212-5p mimic for 2 days. Collagen gel assay was performed after cells reached confluency. Cell contraction (%) was calculated as described in the methods section. *P < 0.05 versus the control mimic group. (**B,C**) After transfection of VSMCs with miR-212-5p mimic, *RhoA* and *Rock2* transcript levels were analyzed using RT-PCR. **P < 0.01 and ***P < 0.001 versus the control mimic group. (**D**) VSMCs were transfected with control or miR-212-5p inhibitor for 2 days, following which a collagen gel assay was performed. *P < 0.05 versus the control inhibitor group. (**E,F**) After transfection of VSMCs with miR-212-5p inhibitor, *RhoA* and *Rock2* transcript levels were analyzed using RT-PCR. **P < 0.01 versus the control inhibitor group. (**G–J**) Representative western blot images of RhoA and ROCK2 expression in miR-212-5p mimic- or inhibitor-transfected VSMCs. β-actin was used as a loading control. Protein levels were quantified using ImageJ software. *P < 0.05 and **P < 0.01 versus the control mimic or inhibitor group.

Vascular contraction responses are increased during hypertension. To investigate whether miR-212-5p plays a role in the regulation of contraction, we performed collagen gel contraction assay. Results showed that transfection with miR-212-5p mimic decreased collagen contraction in response to the thromboxane A2 agonist U46619 (Fig 4A) and reduced the mRNA levels of *RhoA* and *Rock2* in VSMCs (Fig 4B and 4C). In contrast, miR-212-5p inhibitor treatment increased collagen contraction and *RhoA and Rock2* mRNA levels (Fig 4D–4F). miR-212-5p mimic-induced decrease in RhoA and ROCK2 protein expression was further confirmed by western blotting (Fig 4G and 4H). However, opposite results were observed in miR-212-5p inhibitor-transfected cells by western blotting (Fig 4I and 4J).

### miR-212-5p targets PAFAH1B2

**Fig 5:**
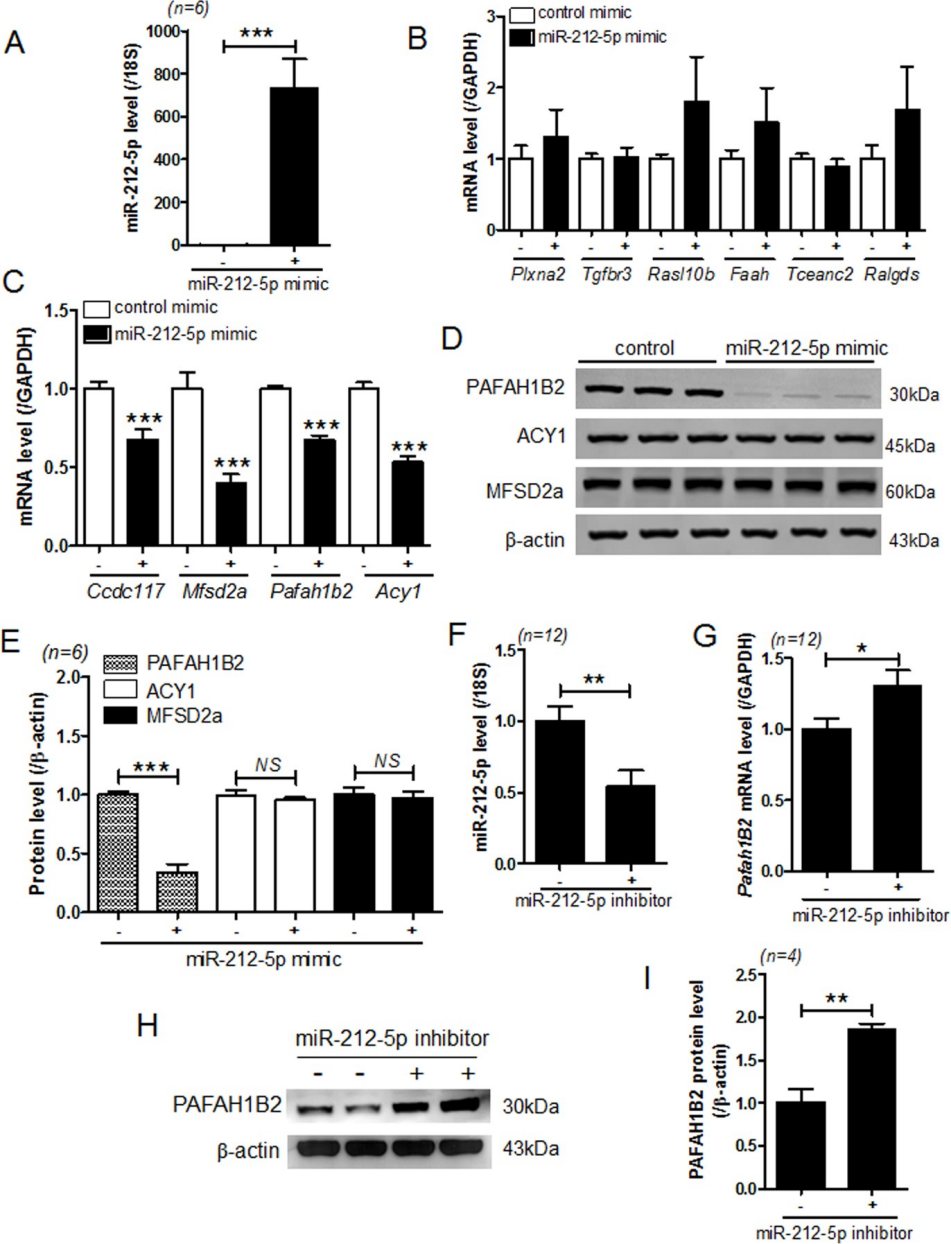
miR-212-5p targets PAFAH1B2. **(A)** VSMCs were transfected with control or miR-212-5p mimic for 24 h, and the expression of miR-212-5p was determined by RT-PCR. ***P < 0.001 versus the control mimic group. **(B,C)** After transfection of VSMCs with miR-212-5p, the candidate target genes of miR-212-5p were analyzed using RT-PCR. ***P < 0.001 versus the control mimic group. **(D,E)** Representative western blot images and quantification. PAFAH1B2, ACY1, and MFSD2a protein levels were assessed in VSMCs after transfection with the control or miR-212-5p mimic. β-actin was used as a loading control. (**F−I**) VSMCs were transfected with control or miR-212-5p inhibitor for 24 h, and the expression of miR-212-5p and its target PAFAH1B2 was assessed by RT-PCR and western blotting. *P < 0.05 and **P < 0.01 versus the control inhibitor group.

To predict the targets of miR-212-5p, we used microRNA target prediction database (miRDB) and tested 10 target genes using RT-PCR. First, we found that transfection with miR-212-5p mimic significantly increased the expression of miR-212-5p in VSMCs (Fig 5A). Among the candidate target genes, the transcript levels of 6 genes were not reduced in miR-212-5p mimic-transfected VSMCs (Fig 5B). These included plexin A2 (*Plxna2*), transforming growth factor beta receptor 3 (*Tgfbr3*), RAS-like family 10 member B (*Rasl10b*), fatty acid amide hydrolase (*Faah*), transcription elongation factor A N-terminal and central domain containing 2 (*Tceanc2*), and ral guanine nucleotide dissociation stimulator (*Ralgds*). On the other hand, coiled-coil domain containing 117 *(Ccdc117*), major facilitator superfamily domain containing 2A (*Mfsd2a*), *Pafah1b2*, and aminoacylase 1 (*Acy1*) expression was significantly reduced in miR-212-5p mimic-transfected VSMCs compared with that in control mimic-transfected cells (Fig 5C). Moreover, transfection with miR-212-5p mimic decreased the protein level of PAFAH1B2, but not MFSD2A and ACY1 (Fig 5D and 5E). Conversely, miR-212-5p inhibitor treatment significantly decreased the expression of miR-212-5p (Fig 5F) and increased the mRNA and protein levels of PAFAH1B2 (Fig 5G–5I).

### PAFAH1B2 protein is distributed in the cytoplasm of VSMCs

**Fig 6:**
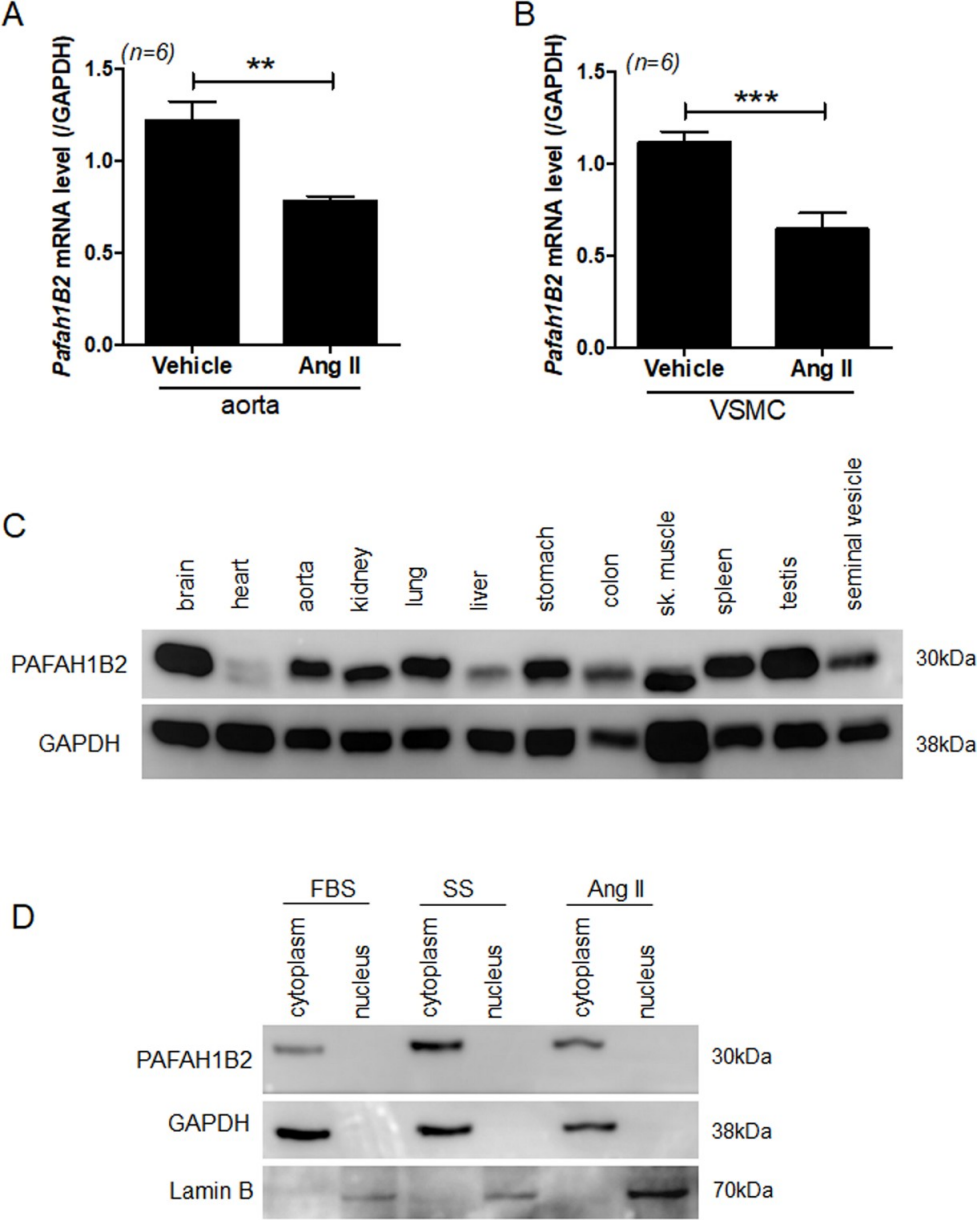
PAFAH1B2 is ubiquitously expressed in most tissues and distributed in VSMC cytoplasmic fraction. **(A)** The mRNA levels of aortic PAFAH1B2 were determined in vehicle- and angiotensin II-treated mice. **P < 0.01 versus the vehicle group. **(B)** VSMCs were serum-starved and treated with angiotensin II (1 μM). The expression of PAFAH1B2 was evaluated using RT-PCR. ***P < 0.001 versus the vehicle group. (**C**) Tissue distribution of PAFAH1B2 in 10-week-old male rats. GAPDH was used as a loading control. (**D**) Representative western blot images of PAFAH1B2 expression in cytoplasmic extracts. Lamin B and GAPDH were used as nuclear and cytoplasmic marker proteins, respectively. FBS indicates that VSMCs were maintained in complete medium; SS indicates serum-starved; and Ang II indicates angiotensin II treatment (1 day).

The expression of the miR-212-5p target gene PAFAH1B2 was investigated in the aorta of angiotensin II-treated mice and angiotensin II-treated VSMCs. The mRNA expression of *Pafah1B2* was decreased in angiotensin II-treated mice (Fig 6A) as well as VSMCs (Fig 6B). Next, we examined the tissue distribution of PAFAH1B2. Tissue blots showed that PAFAH1B2 was ubiquitously expressed in all tissues, including the aorta (Fig 6C). To further determine the subcellular distribution of PAFAH1B2, western blot analysis was performed using nuclear and cytoplasmic extracts from VSMCs. PAFAH1B expression was only detected in the cytoplasmic extracts (Fig 6D, the most left panel). Even under angiotensin II stimulation, PAFAH1B2 was not translocated to the nucleus (Fig 6D).

### PAFAH1B2 regulates cell proliferation and contraction

**Fig 7:**
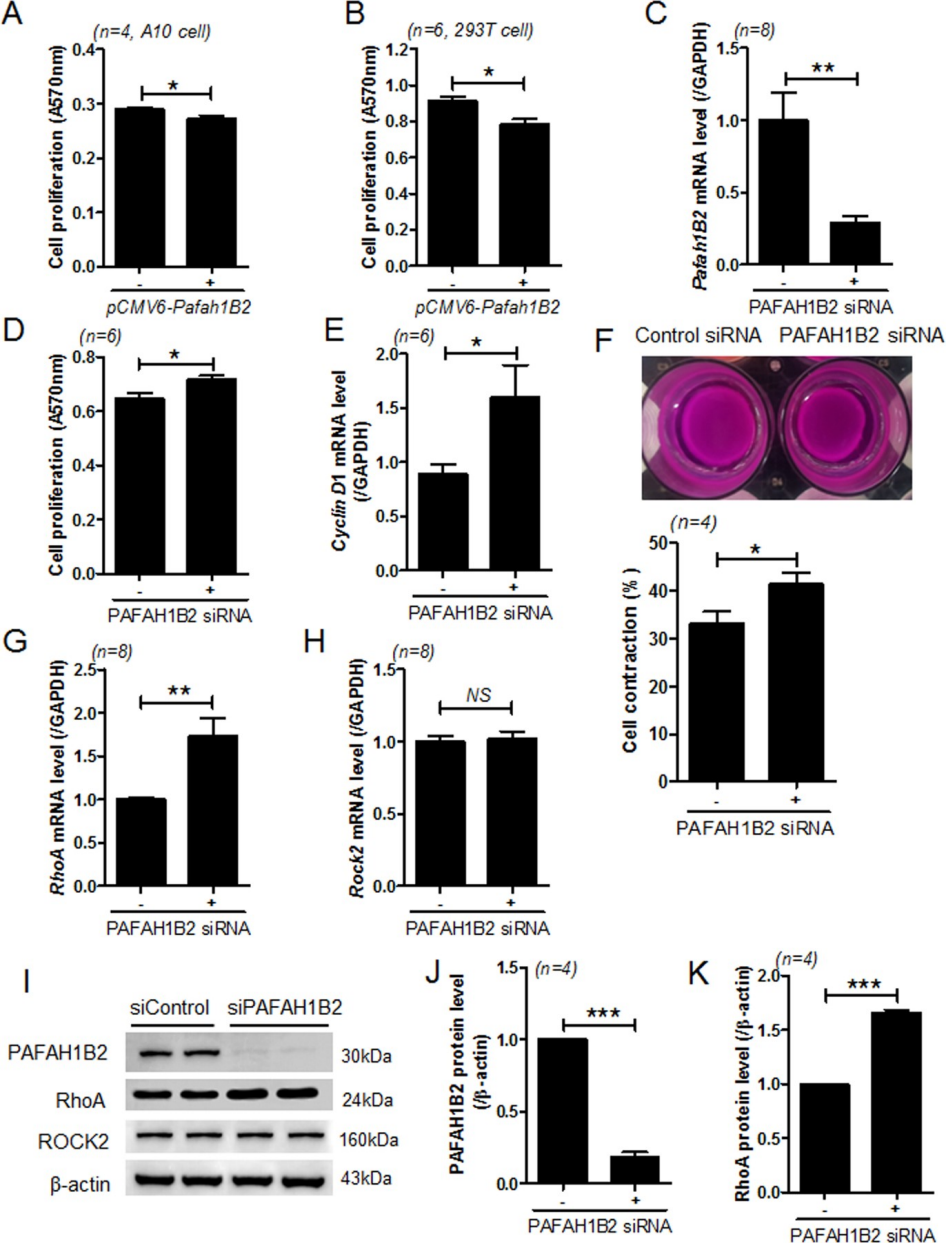
PAFAH1B2 regulates VSMC proliferation and contraction. **(A,B)** A10 or 293T cells were transfected with empty vector or *pCMV6-Pafah1b2*, and cell proliferation was evaluated by the MTT assay. *P < 0.05 versus the vector-transfected group. **(C)** Transfection of VSMCs with PAFAH1B2 siRNA reduced the endogenous PAFAH1B2 mRNA levels. **P < 0.01 versus the control siRNA group. **(D)** VSMCs were transfected with control or PAFAH1B2 siRNA, and cell proliferation was evaluated by the MTT assay. *P < 0.05 versus the control siRNA group. **(E)** After transfection of VSMCs with PAFAH1B2 siRNA, *cyclin D1* transcript levels were determined using RT-PCR. *P < 0.05 versus the control siRNA group. (**F**) VSMCs were transfected with control or PAFAH1B2 siRNA for 2 days, and then, collagen gel assay was performed. *P < 0.05 versus the control siRNA group. **(G,H)** After transfection of VSMCs with PAFAH1B2 siRNA, *RhoA and Rock2* transcript levels were determined using RT-PCR. **P < 0.01 versus the control siRNA group. (**I**) Representative western blot images of RhoA and ROCK2 expression in PAFAH1B2 siRNA-transfected VSMCs. (**J,K**) Protein levels were quantified using ImageJ software. *P < 0.05 and **P < 0.01 versus the control siRNA group.

We investigated whether PAFAH1B2 affects cell proliferation. Transfection of A10 and 293 T cells with *pCMV6-Pafah1b2* decreased cell proliferation (Fig 7A and 7B). Next, we observed that transfection VSMCs with PAFAH1B2 siRNA successfully downregulated *Pafah1B2* mRNA levels (Fig 7C). However, knockdown of PAFAH1B2 increased VSMC proliferation (Fig 7D) and *cyclin D1* mRNA levels (Fig 7E). Furthermore, we investigated the role of PAFAH1B2 in vascular contractility. Collagen gel assay revealed that knockdown of PAFAH1B2 increased vascular contraction (Fig 7F). In addition, PAFAH1B2 knockdown significantly increased the mRNA levels of *RhoA* (Fig 7G) but not those of *Rock2* (Fig 7H), decreased the PAFAH1B2 protein level (Fig 7I and 7J), and increased the RhoA protein level (Fig 7K).

## Discussion

Angiotensin II promotes VSMC growth and arterial remodeling in hypertension [26]. The expression of several miRNAs is altered in response to angiotensin II in mice. In the present study, we demonstrated the negative role of miR-212-5p in VSMC proliferation, migration, and contraction. Our results showed that overexpression of miR-212-5p inhibited VSMC proliferation, migration, and collagen gel contraction, whereas knockdown of miR-212-5p exerted opposite effects, indicating that miR-212-5p acts as an endogenous negative regulator of VSMC growth and contraction.

The expression of miR-212-5p was significantly increased in the mouse aortic tissues and VSMCs after angiotensin II treatment. However, miR-212-3p expression was not significantly increased by angiotensin II treatment as determined by RT-PCR analysis. In accordance with previous findings, our microarray results also showed that miR-34c-5p and miR-34c-3p expression was most highly induced in response to angiotensin II treatment [27,28]. Moreover, miR-34c and miR-34a have been reported to inhibit VSMC proliferation by targeting stem cell factor and neurogenic locus notch homolog protein-1, respectively [19,29]. Hence, we further investigated the role of miR-212-5p in this study. Stimulation with angiotensin II may induce the expression of miRNAs that promote or inhibit cell proliferation. Notably, an increase in miR-212-5p expression in angiotensin II-treated mice was shown to suppress cell proliferation.

miR-212/132 is a well-known gene cluster located on chromosome 11 in mice [30]. Both miR-212 and -132 play a role in inhibiting cell proliferation and migration in VSMCs [31] but exert differential effects on endothelial transcriptome regulation [32]. miRNAs have multiple target genes. miR-212-5p was shown to suppress epithelial-mesenchymal transition (EMT) in aggressive breast cancer by targeting paired related homeobox2 [24]. Additionally, miR-212-5p acts as a tumor-suppressor gene in acute myeloid leukemia by targeting frizzled class receptor 5 [33]. Distinct miRNAs can regulate cell proliferation under different environments. For example, upregulation of miR-21, miR-214, and miR-352 was detected in the vascular wall after angioplasty [34]. In our study, the expression of miR-34c-5p, miR-34b-3p, miR-34c-3p, miR-301a-3p, miR-212-3p, miR-381-3p, miR-127-5p, miR-409-5p, and miR-212-5p was upregulated in the aorta of angiotensin II-induced hypertension mice.

Next, we identified PAFAH1B2 as an effective downstream target of miR-212-5p. An association between plasma PAF-AH gene R92H polymorphisms and coronary heart diseases has been previously reported [35]. Moreover, elevated serum levels of platelet-activating factor and PAFAH are associated with high risk of systemic vasculitis Kawasaki disease [36]. PAFAH1B2 is the catalytic subunit of PAF-AH and has been reported to be overexpressed in several cancers [37]. PAFAH1B2 also regulates the process of EMT in pancreatic ductal adenocarcinoma [38]. However, PAFAH1B2 is likely to act as a dual player depending on the pathological environment. Some studies have shown that PAFAH1B2 plays a role in promoting cancer, and studies are underway for the development of drugs that inhibit PAFAH1B2 [39]. In contrast, our study suggests that PAFAH1B2 plays a role in inhibiting VSMC proliferation and contraction in hypertension.

MFSD2A, another target of miR-212-5p, has been shown to have beneficial effects in gastric cancer [40]. In addition, MFSD2A was shown to reduce colitis in mice through ω-3 docosahexaenoic acid-induced metabolism in the gut vasculature [41]. Both PAFAH1B1 and MFSD2A, targets of miR-212-5p, have similar roles in cell proliferation. In the present study, we found that PAFAH1B2 was ubiquitously expressed in almost all mouse tissues, but only distributed in the cytoplasm of VSMCs. The RhoA-ROCK pathway has been reported to be associated with vascular contraction [42]. Interestingly, in our study, knockdown of PAFAH1B2 increased vascular contraction, as well as the mRNA and protein levels of RhoA in VSMCs. Similarly, RhoA has been shown to be mainly expressed in the cytosol [43]. These results suggest that PAFAH1B2 and RhoA may contribute to vascular contraction. In addition, the RhoA-ROCK pathway has been known to be a key regulator of cell proliferation and migration [44]. Therefore, our results demonstrate that miR-212-5p and its target PAFAH1B2 are involved in the regulation of cell proliferation, migration, and contraction via modulation of RhoA expression in the RhoA-ROCK pathway.

The limitation of our study is the possibility that other miR-212-5p targets could play a role opposite to PAFAH1B2. For example, Ccdc117, another target of miR-212-5p, is involved in the regulation of cyclin E by promoting the proliferation of HeLa cells [45]. ACY1 is also a target of miR-212-5p and has been reported to increase the proliferation of colorectal cancer HT-29 cells [46]. However, both Ccdc117 and ACY1 primarily appear to play a role in cancers, which are a group of diseases that are mechanistically different from vascular disease.

In conclusion, our results suggested miR-212-5p as a novel miRNA that inhibits VSMC proliferation in angiotensin II-induced hypertension. Here, miR-212-5p and its target PAFAH1B2 were shown to inhibit VSMC proliferation and migration as well as vascular contraction. Hence, overexpression of miR-212-5p and PAFAH1B2 may serve as a potential therapeutic strategy for hypertension.

## Supporting information

S1 File (PDF)
