# Supplementary material for: MicroRNA-212-5p and its target PAFAH1B2 suppress vascular proliferation and contraction via the downregulation of RhoA
Source: PLoS One. 2021 Mar 24;16(3):e0249146. doi: 10.1371/journal.pone.0249146 (PMC7990166; doi:10.1371/journal.pone.0249146)

Figure 4G Original WB blots

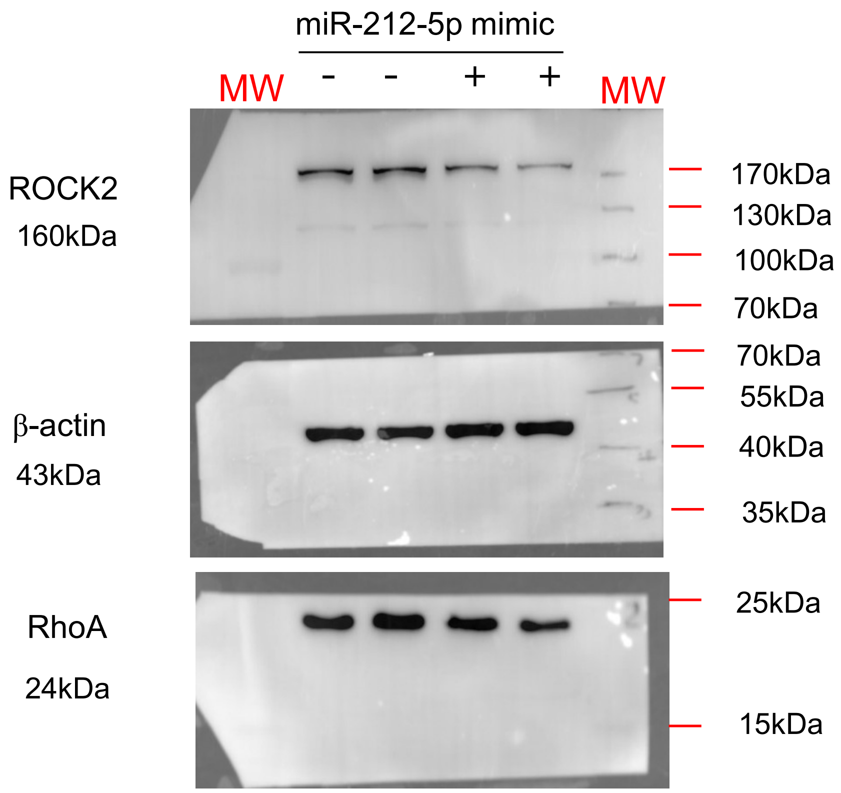

Figure 4I Original WB blots

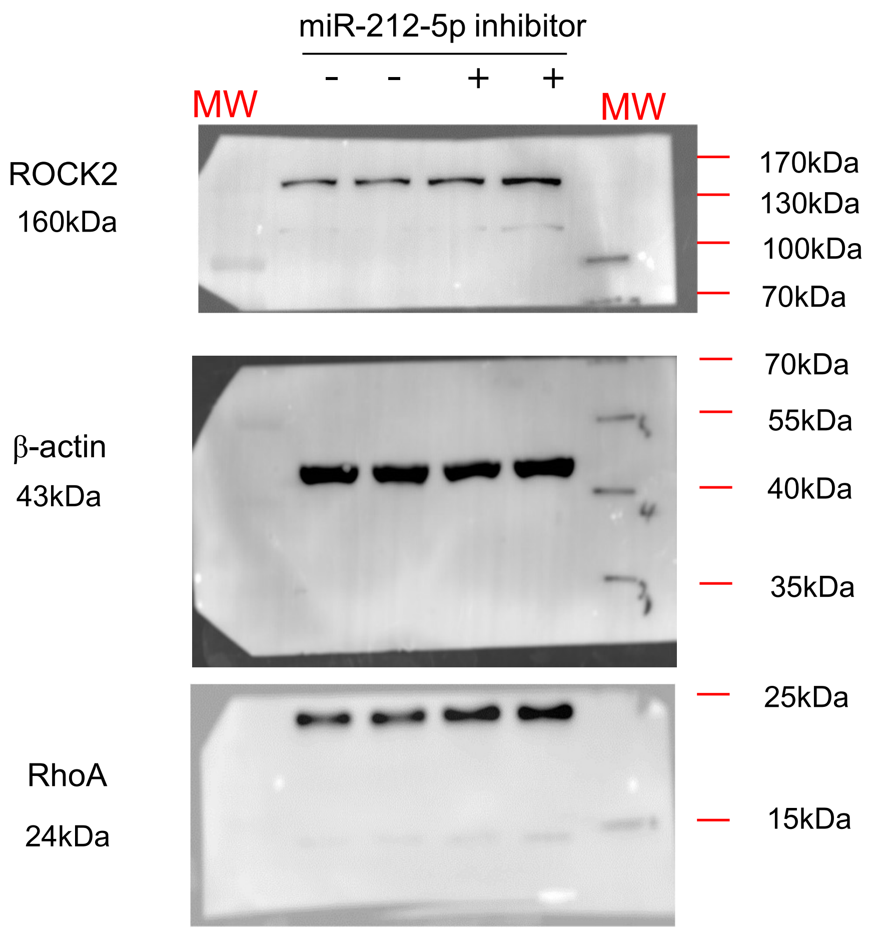

Figure 5D Original WB blots

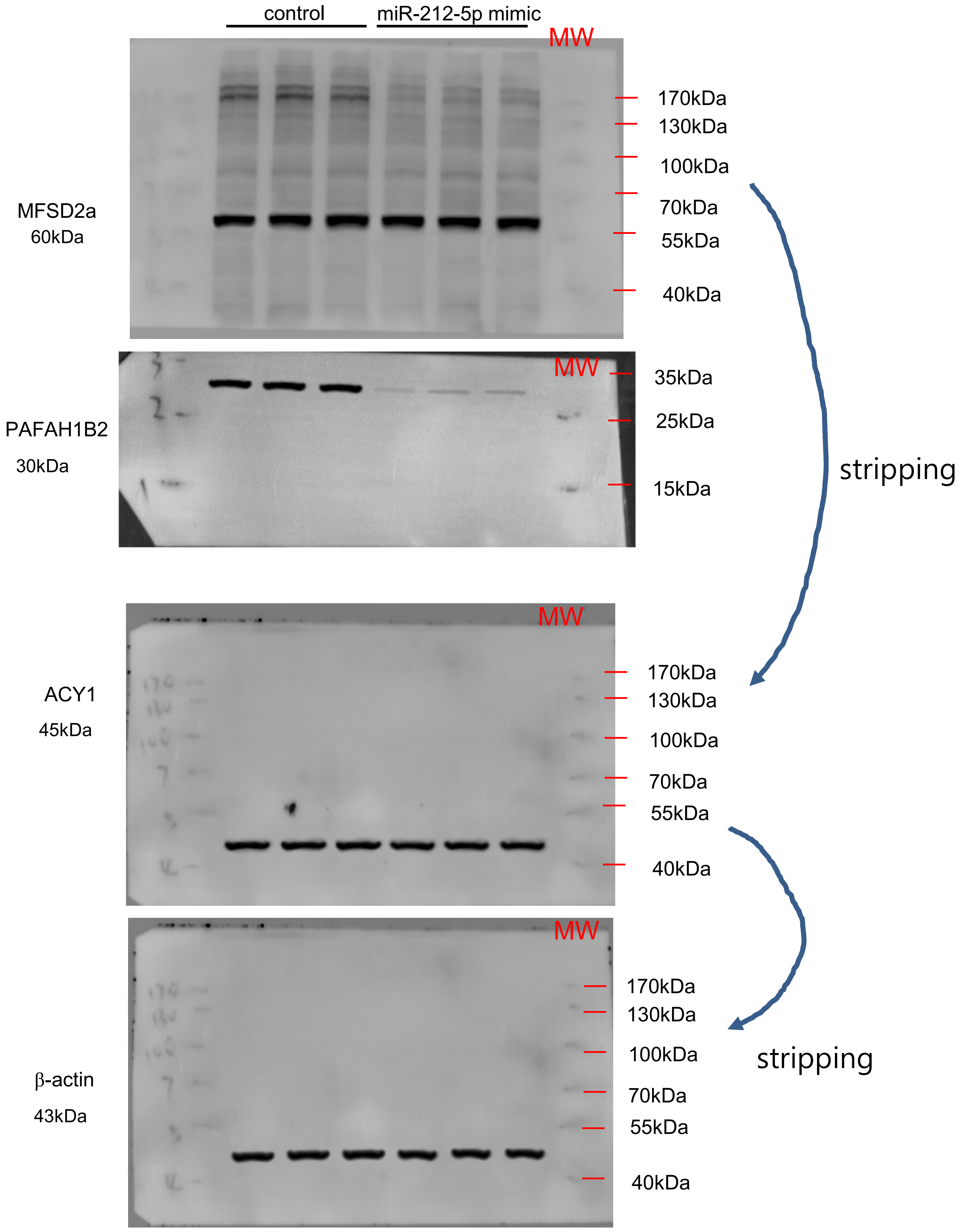

Figure 5H Original WB blots

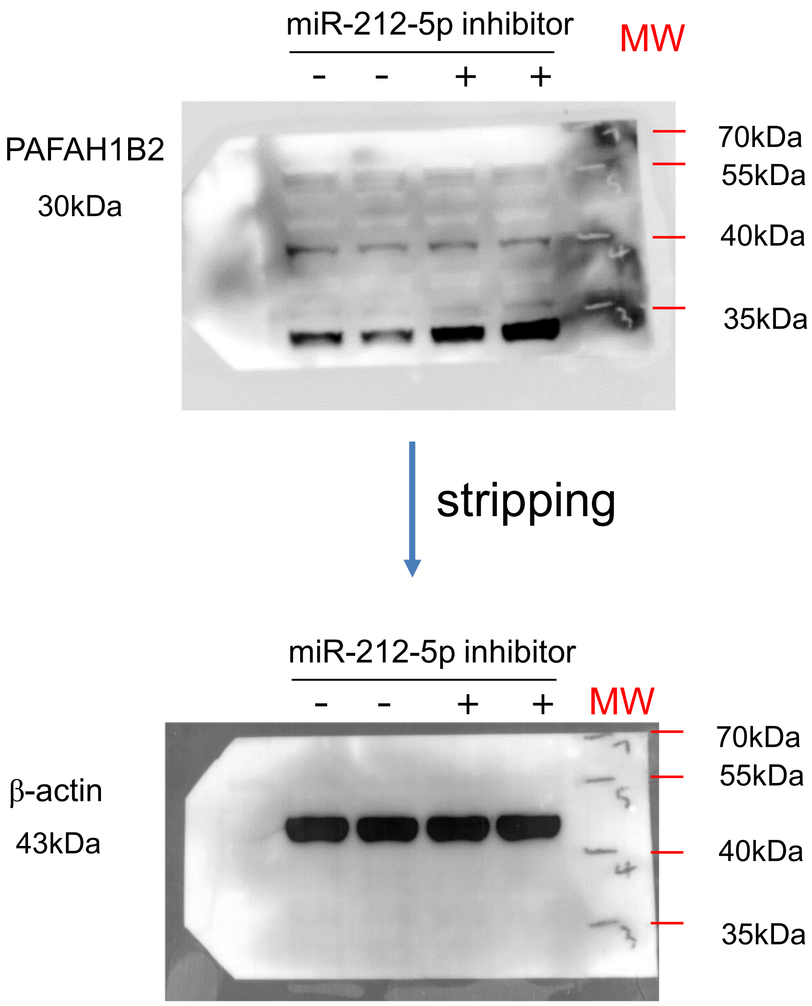

Figure 6C Original WB blots

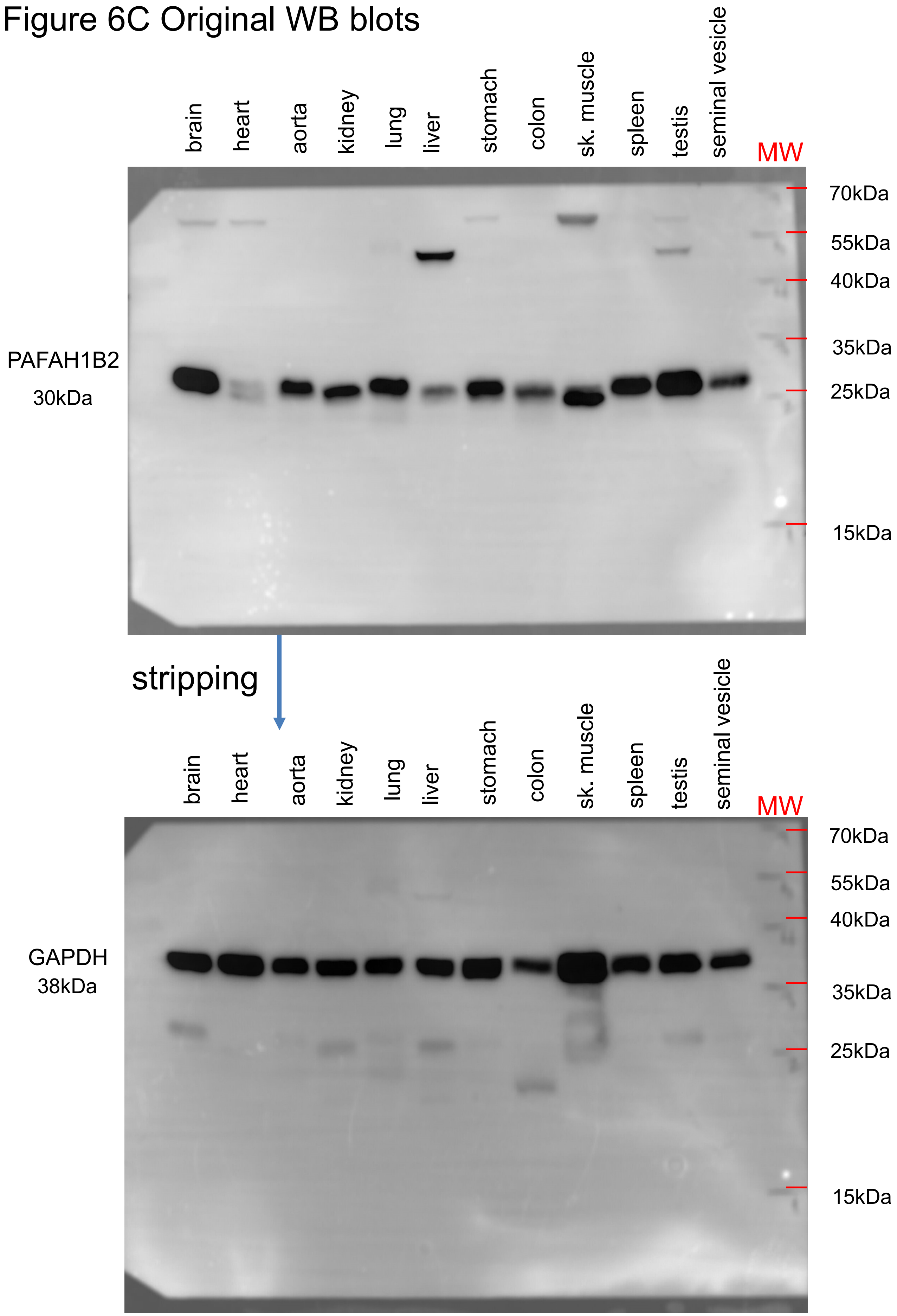

Figure 6D Original WB blots

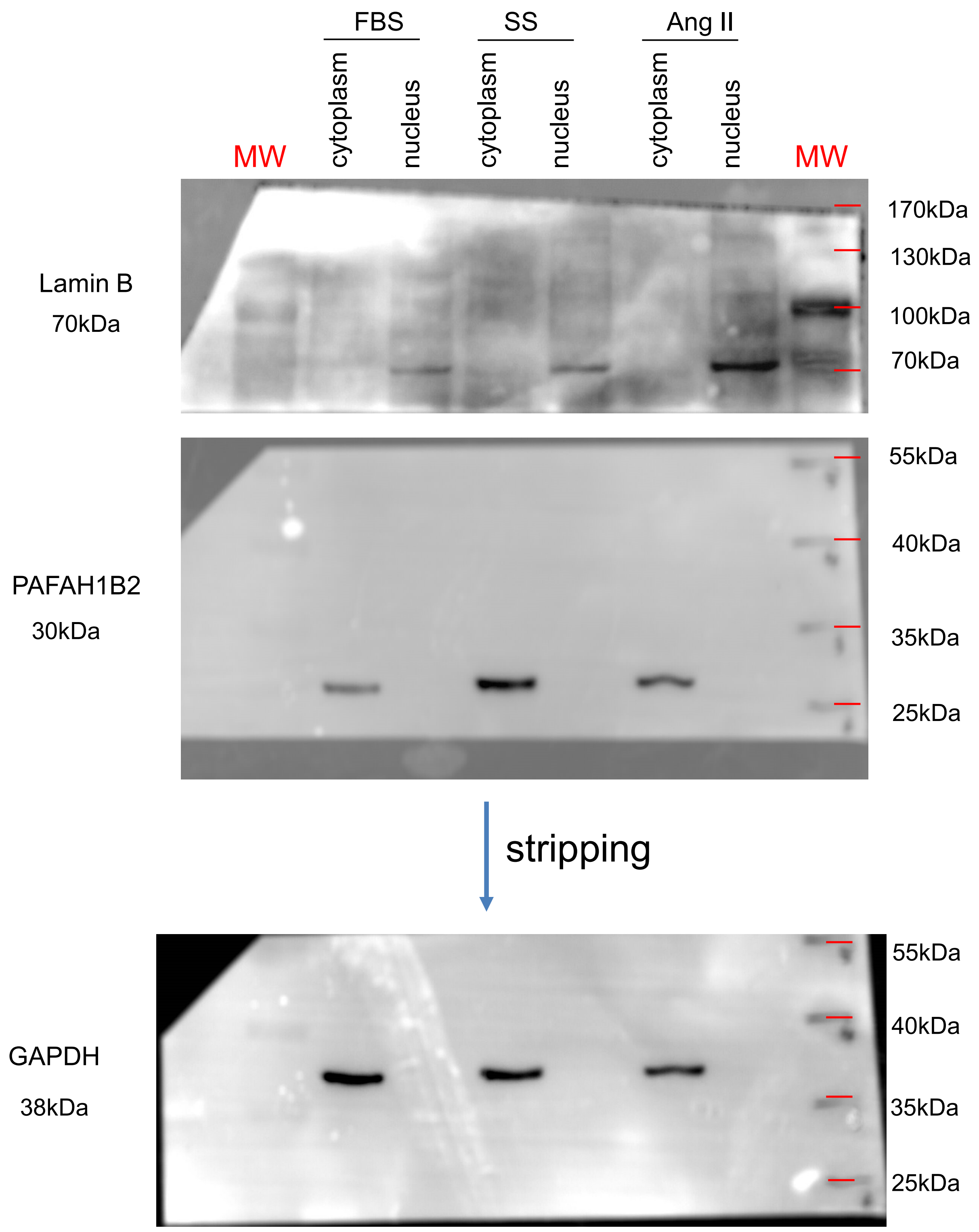

Figure 7I Original WB blots

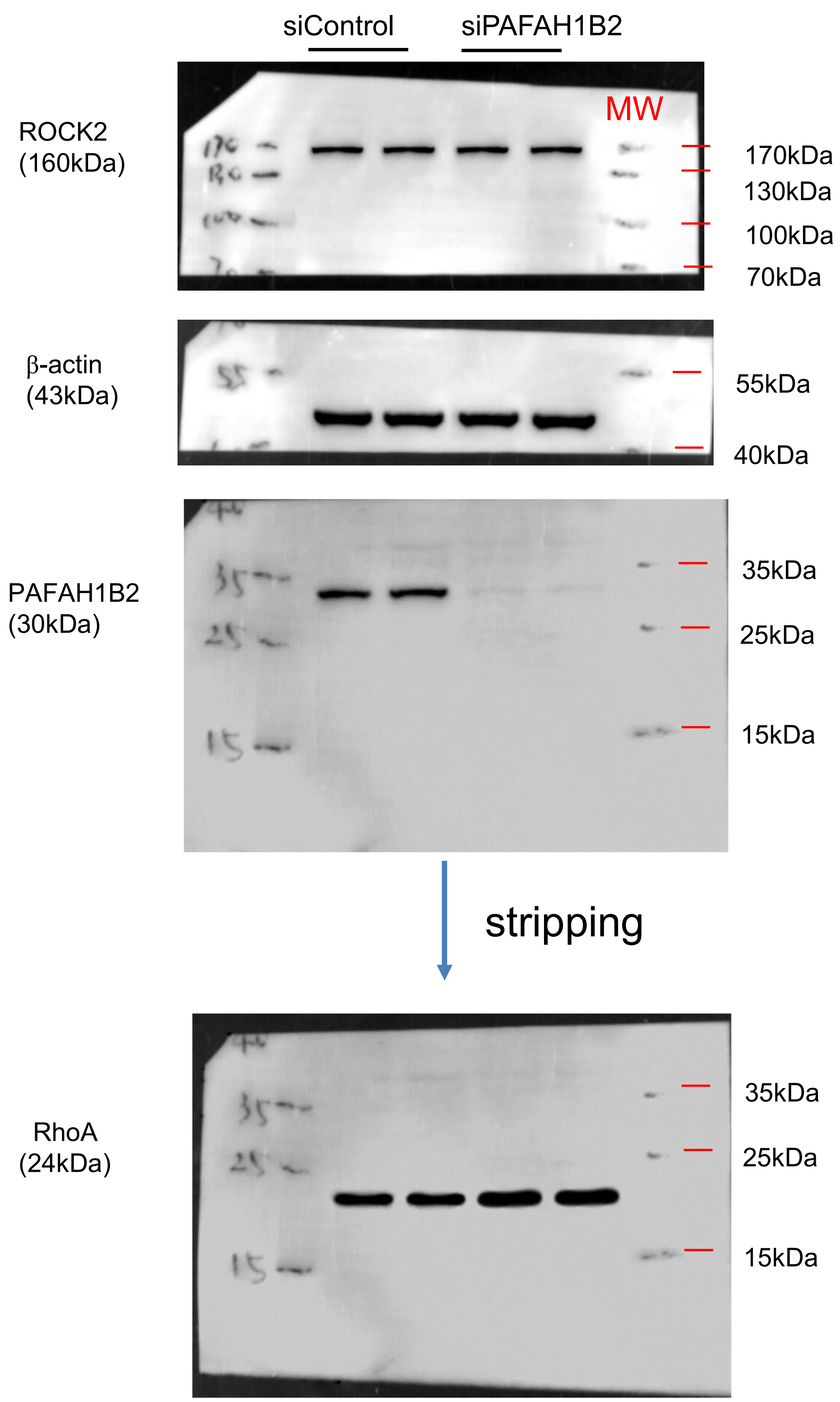

Supplement: S1 File — (PDF) [file pone.0249146.s001.pdf]
